# Effects of Intermittent Fasting in Human Compared to a Non-intervention Diet and Caloric Restriction: A Meta-Analysis of Randomized Controlled Trials

**DOI:** 10.3389/fnut.2022.871682

**Published:** 2022-05-02

**Authors:** Lihu Gu, Rongrong Fu, Jiaze Hong, Haixiang Ni, Kepin Yu, Haiying Lou

**Affiliations:** ^1^Department of General Surgery, HwaMei Hospital, University of Chinese Academy of Sciences, Ningbo, China; ^2^The First Clinical Medical College, Zhejiang Chinese Medical University, Hangzhou, China; ^3^The Second Clinical Medical College, Zhejiang Chinese Medical University, Hangzhou, China; ^4^The Department of Endocrinology, The First Affiliated Hospital of Zhejiang Chinese Medical University, Hangzhou, China; ^5^Department of Endocrinology, Zhuji People's Hospital, Shaoxing, China

**Keywords:** intermittent fasting, non-intervention diet, caloric restriction, effect, meta-analysis

## Abstract

**Background:**

The popularity of applying intermittent fasting (IF) has increased as more and more people are trying to avoid or alleviate obesity and metabolic disease. This study aimed to systematically explore the effects of various IF in humans.

**Methods:**

The randomized controlled trials (RCTs) related to IF vs. non-intervention diet or caloric restriction (CR) were retrieved in PubMed, Web of Science, Cochrane Library database, and Embase. Extraction outcomes included, but were not limited to, weight, body mass index (BMI), waist circumference (WC), fasting glucose, and triglyceride (TG).

**Results:**

This study includes 43 RCTs with 2,483 participants. The intervention time was at least 1 month, and the median intervention time was 3 months. Contrasting results between IF and non-intervention diet showed that participants had lower weight (weighted mean difference (WMD) = 1.10, 95% CI: 0.09–2.12, *p* = 0.03) and BMI after IF (WMD = 0.38, 95% CI: 0.08–0.68, *p* = 0.01). The WC of participants after IF decreased significantly compared with the non-intervention diet (WMD = 1.02, 95% CI: 0.06–1.99, *p* = 0.04). IF regulated fat mass (FM) more effectively than non-intervention diet (WMD = 0.74, 95% CI: 0.17–1.31, *p* = 0.01). The fat-free mass of people after IF was higher (WMD = −0.73, 95% CI: (−1.45)–(−0.02), *p* = 0.05). There was no difference in fasting blood glucose concentrations between participants in the after IF and non-intervention diet groups. The results of insulin concentrations and HOMA-IR, though, indicated that IF was significantly more beneficial than non-intervention diet (standard mean difference (SMD) = −0.21, 95% CI: 0.02–0.40, *p* = 0.03, and WMD = 0.35, 95% CI: 0.04–0.65, *p* = 0.03, respectively). Cholesterol and TG concentrations in participants after IF were also lower than that after a nonintervention diet (SMD = 0.22, 95% CI: 0.09–0.35, *p* = 0.001 and SMD = 0.13, 95% CI: 0.00–0.26, *p* = 0.05, respectively). IF outcomes did not differ from CR except for reduced WC.

**Conclusion:**

Intermittent fasting was more beneficial in reducing body weight, WC, and FM without affecting lean mass compared to the non-intervention diet. IF also effectively improved insulin resistance and blood lipid conditions compared with non-intervention diets. However, IF showed less benefit over CR.

## Introduction

Recently, the number of patients with metabolic diseases and obesity around the world has increased significantly (1). Numerous treatments for these kinds of diseases mostly focus on diet and exercise (2, 3). Intermittent fasting (IF) has become a popular lifestyle in recent years, though it has existed in religious and cultural contexts for a long time (4, 5). IF is a term that covers several specific patterns (6, 7). Alternate-day fasting (ADF), intermittent energy restriction (IER), time-restricted feeding (TRF), and Ramadan fasting are currently recognized as the several subtypes of IF (7, 8). ADF consists of alternating fasting days and eating days, while TRF refers to the daily free energy intake within a specific time window of 4–12 h (8). Ramadan fasting refers to one meal only before dawn or after sunset (8, 9). There is also a modified fasting regime that differs from the previous ones in terms of not taking in any energy during the fasting period and eating food with extremely low calories 2 or 3 days per week (8).

Intermittent fasting is a specific strategy to reduce energy intake *via* fasting and, therefore, reduce weekly energy intake. It has been an attemption in patients with overweight or obese to achieve weight loss (6). Caloric restriction (CR), which is considered the standard dietary strategy to lose or maintain weight, has been discovered to be difficult in maintaining among many individuals, and the likelihood of late weight rebound when it comes to long-term use. Thus, a combination of intermittent CR and temporal control was proposed to replace a simple long-term regimen of CR (10, 11).

A meta-analysis reported that ADF can result in better compliance and more reduced fat mass (FM) than CR in patients (11). Furthermore, a recent investigation into TRF has illustrated its effectiveness in weight loss (12). Although some studies have revealed the effects of certain fasting patterns, comprehensive systematic reviews of IF are limited and in need. Therefore, herein, we attempted to explore the effects of IF on humans systematically and quantitatively.

## Methods

### Literature Search Strategy

Two researchers independently screened the literature related to IF from PubMed, Web of Science, Cochrane Library database, and Embase. All selected literature was published before June 2021. The keywords used in the search were “randomized controlled trial” OR “controlled clinical trial” OR “randomized” OR “placebo” OR “clinical trials as topic OR randomly” OR “trial” AND “fasting” OR “intermittent^*^ fast^*^” OR “fast^*^ diet^*^” OR “alternat^*^ fast^*^” OR “modified fast^*^” OR “food abstinence” OR “food fast^*^” OR “diet^*^ restricti^*^” OR “food restricti^*^” OR “time restricted feed^*^” OR “time restricted fast^*^” OR “time-restricted eating” OR “one whole day fast^*^” OR “food tim^*^” OR “Ramadan” OR “Ramadhan” OR “Ramadan fasting” OR “Islamic fasting” OR “Ramadan intermittent fasting” OR “Ramadan diurnal fasting” OR “Ramadan model of intermittent fasting” OR “intermittent prolonged fasting during Ramadan” OR “Ramadan fast” OR “recurrent circadian fasting.” The references in the relevant literature were screened additionally to expand the library of relevant literature. The study guideline was Preferred Reporting Items for Systematic Review and Meta-Analysis (PRISMA) (13).

### Inclusion Criteria and Exclusion Criteria

Literature meeting all the following criteria would be included: (1) The intervention group included but was not limited to ADF, TRF, IER, and Ramadan fasting, while the control group was the non-intervention diet or CR. (2) The study provided physical and biochemical parameters of the subjects in both the intervention group and the control group after intervention. (3) The follow-up time was at least 1 month. (4) Data were available.

The study would be excluded once it met any of the following criteria: (1) The study was not a randomized controlled trial (RCT). (2) The research individual was not human. (3) The full text of the article was not in English. (4) The literature was duplicated.

### Quality Assessment and Data Extraction

The quality of screened RCTs was evaluated using the Cochrane Collaboration's tool (14). Two investigators independently assessed the risk of study bias. Controversies were decided by a third researcher. Study characteristics (author, year, country, trial registration, patient inclusion criteria, intervention, control, and follow-up) and physical parameters [weight, body mass index (BMI), waist circumference (WC)], biochemical parameters [fasting insulin, fasting glucose, total cholesterol (TC), and triglyceride (TG)] were extracted from the included studies.

Intermittent fasting patterns were diverse, and the current major dietary patterns were ADF, TRF, IER, and Ramadan fasting (7, 8). This study was roughly divided into daily TRF, weekly TRF, ADF, and Ramadan fasting based on the time and frequency of fasting with IF. Among these, daily TRF referred to a feeding window of 4–12 h per day and fasting for the remaining of the day. Weekly TRF referred to fasting for 2–3 days per week consisting of a 5:2 diet and a 3:4 diet. Control group dietary patterns were diverse, including groups following uncontrolled habitual “non-intervention diets,” “calorie restriction,” “continuous energy restriction,” and “daily energy restriction” groups: the last three are referred to in this study as CR.

### Statistical Analysis

Review Manager 5.3 was used for statistical analysis. The weighted mean difference (WMD) and the standard mean difference (SMD) were calculated for continuous data. The 95% confidence intervals (CIs) for all statistical results were provided. *I*^2^ was used to represent the heterogeneity of the results. When *I*^2^ > 50, the random-effect model was used, otherwise, the fixed-effect model was used. *p* < 0.05 was considered to be statistically significant. Sensitivity analysis was performed by removing the included studies one by one. Meanwhile, funnel plots were used to examine publication bias (15).

## Results

### Study Characteristics

**Figure 1:**
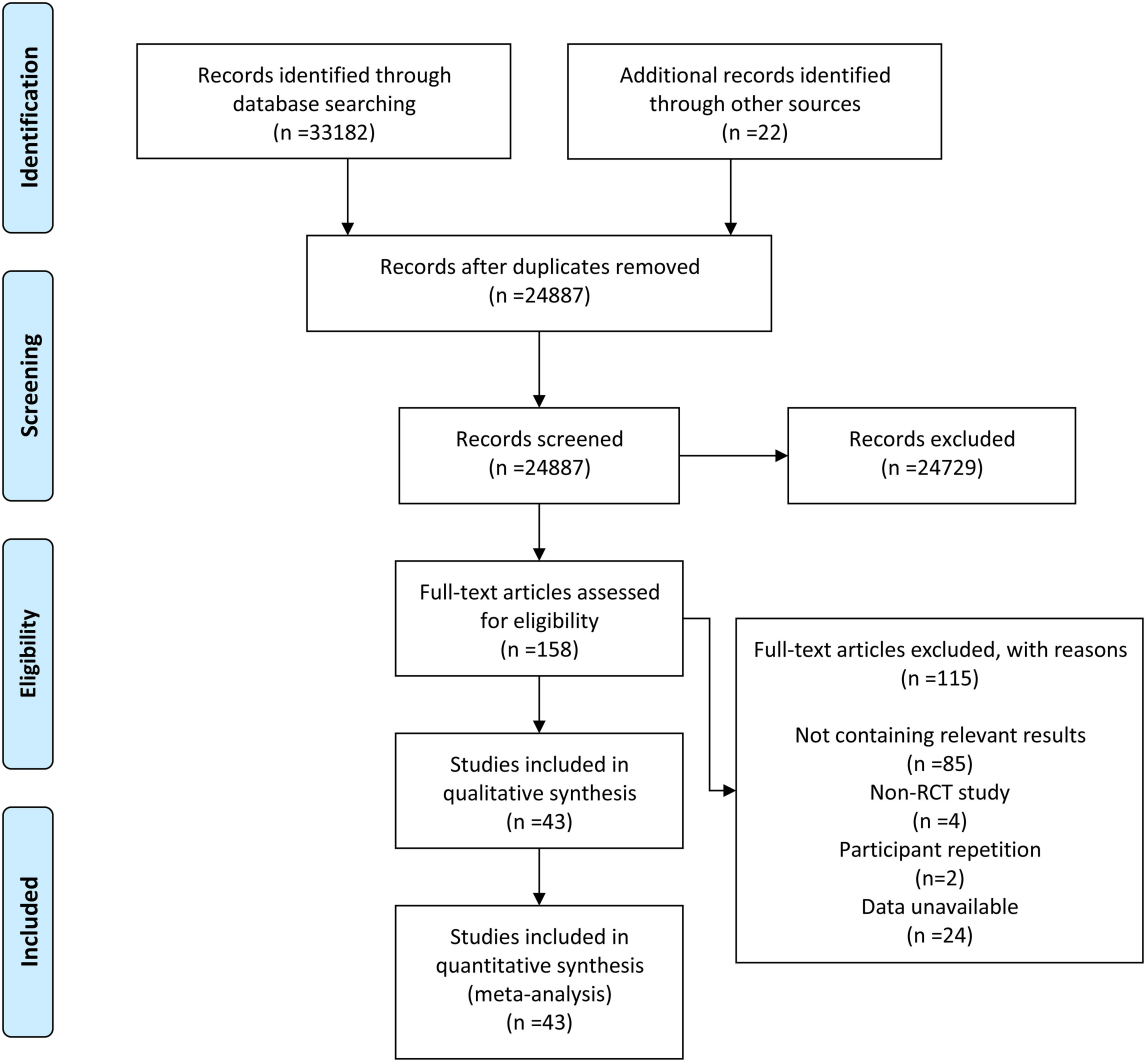
The flowchart for the retrieval process included studies in this meta-analysis.

After retrieval and elimination of duplicates, a total of 24,887 articles were screened. Then, 24,729 of them were excluded based on their irrelevant title and abstract. After thoroughly viewing the full text of the remaining 158 articles, 115 of them were excluded, of which 85 were excluded due to lack of relevant results, 4 exclusions were due to non-RCTs, 2 were excluded due to population duplication, and 24 were excluded due to unavailable data. Finally, 43 RCTs (3, 6, 7, 9, 16–54) were included in our meta-analysis. The detailed retrieval process and elimination reasons are recorded in the flowchart (Figure 1).

Characteristics of included RCTs in the meta-analysis were recorded in Table 1. A total of 2,483 participants were included in the analysis, with 1,277 in the intervention group and 1,206 in the control group. Interventions included weekly TRF, daily TRF, ADF, and Ramadan fasting. Eating patterns in the control group included non-intervention diet and CR. Non-intervention diet referred to the usual diet without any intervention in the subjects. CR included continuous energy restriction, a Mediterranean diet, and Dietary Approaches to Stop Hypertension (DASH). The included studies were followed for at least 1 month, with a median follow-up of 3 months. Participants in this study came from Brazil, China, Germany, Iran, Italy, Korea, Malaysia, New Zealand, Norway, Spain, Tunisia, Turkey, the UK, and the USA.

**Table 1:** Characteristics of included RCTs in the meta-analysis.

**References**	**Country**	**Trial registration**	**Patient inclusion criteria**	**Intervention**		**Control**		**Follow-up (month)**
				**Fasting patterns**	**N**	**Dietary patterns**	**N**	
Antoni et al. (16)	UK	ISRCTN13687043	BMI>25 kg/m^2^; Age: 18–65 years	IER (2:5 diet)	24	CER	24	16
Beaulieu et al. (17)	UK	NCT03447600	BMI: 25–34.9 kg/m^2^; Age: 18–55 years	ADF	24	CER	22	10
Bhutani et al. (18)	USA	NA	BMI: 30–39.9 kg/m^2^; Age: 25–65 years	ADF	25	Non-intervention diet	16	3
Bowen et al. (19)	Australia	ACTRN12616000110482	BMI > 27 kg/m^2^; Age: 25–60 years	ADF+DER	82	DER	81	4
Cai et al. (20)	China	ChiCTR1900024411	BMI > 24 kg/m^2^ with NAFLD; Age: 18–65 years	ADF	95	Non-intervention diet	79	3
				TRF (16:8 diet)	97			
Carlson et al. (21)	USA	NA	BMI: 18–25 kg/m^2^; Age: 40–50 years	TRF (20:4 diet)	15	Non-intervention diet	15	4
Catenacci et al. (22)	USA	NA	BMI ≥ 30 kg/m^2^; Age: 18–55 years	ADF	15	CR	14	8
Chow et al. (23)	USA	NCT03129581	BMI ≥ 25 kg/m^2^; Age: 18–65 years	TRF (16:8 diet)	13	Non-intervention diet	9	3
Cienfuegos et al. (24)	USA	NCT03867773	BMI: 30–49.9 kg/m^2^; Age: 18–65 years	TRF (20:4 diet)	19	Non-intervention diet	19	2
				TRF (18:6 diet)	20			
Conley et al. (25)	Australia	ACTRN12614000396628	BMI ≥ 30 kg/m^2^; Age: 55–75 years	IER (2:5 diet)	12	CR	12	6
Corley et al. (26)	New Zealand	ACTRN12614000402640	BMI: 30–45 kg/m^2^ with T2DM; Age > 18 years	TRF (2:5 diet)	22	CR	19	4
Correia et al. (7)	USA	NA	Age: 18–30 years	TRF (16:8 diet)	9	Non-intervention diet	9	1
Coutinho et al. (27)	Norway	NCT02169778	BMI: 30–40 kg/m^2^; Age: 18–65 years	IER (3:4 diet)	18	CER	17	3
de Oliveira Maranhão Pureza et al. (44)	Brazil	NA	BMI: 30–45kg/m^2^	TRF+CR (12:12 diet)	31	CR	27	12
Gabel et al. (28)	USA	NCT00960505	BMI: 25–39.9 kg/m^2^; Age: 18–65 years	ADF	34	CR	35	12
						Non-intervention diet	31	
Guo et al. (29)	China	NCT03608800	Age: 30–50 years with comorbidities	IF (2:5 diet)	23	Non-intervention diet	23	2
Harvie et al. (30)	UK	NA	BMI: 24–40kg/m^2^; Age: 30–45 years	IER (2:5 diet)	53	CER	54	6
Harvie et al. (31)	USA	ISRCTN52913838	BMI: 24–45 kg/m^2^; Age:20–65 years	IECR (2:5 diet)	37	DER	40	16
Headland et al. (3)	Australia	ACTRN12614001041640	BMI ≥ 27 kg/m^2^; Age>18 years	IER (2:5 diet)	82	CER	81	13
Hirsh et al. (32)	USA	NCT03372109	BMI: 25–29.9 kg/m^2^; Age: 21–65 years	IF (2:5 diet)	10	Non-intervention diet	12	2
Kotarsky et al. (33)	USA	NCT03823872	BMI: 25–34.9 kg/m^2^; Age: 35–65 years	TRF (16:8 diet)	13	Non-intervention diet	10	14
Kunduraci et al. (34)	Turkey	NCT04502329	BMI ≥ 27 kg/m^2^ with comorbidities; Age: 18–65 years	IER (16:8 diet)	35	CER	35	3
Lowe et al. (35)	USA	NCT03393195	Age: 18–64	TRF (16:8 diet)	69	Non-intervention diet	72	3
Martens et al. (35)	USA	NCT02970188	BMI <40 kg/m^2^; Age: 55–79 years	TRF (16:8 diet)	24	CR	24	2
Martínez-Rodríguez et al. (6)	Spain	NCT04404413	NA	IF	14	Non-intervention diet	14	4
McAllister et al. (37)	USA	NA	NA	TRF (16:8 diet)	11	Non-intervention diet	12	1
Moro et al. (38)	Italy	NA	NA	TRF (16:8 diet)	17	Non-intervention diet	17	2
Moro et al. (39)	Italy	NCT04320784	Age <23 years	TRF (16:8 diet)	8	Non-intervention diet	8	1
Oh et al. (40)	Korea	NCT03652532	BMI > 23 kg/m^2^; Age: 18–64 years	ADF+CR	13	CR	10	2
Panizza et al. (41)	USA	NCT03639350	BMI: 25–40 kg/m^2^; Age: 35–55 years	IER+MED (2:5 diet)	30	DASH	30	6
Parvaresh et al. (42)	Iran	IRCT201509092395N8	BMI: 25–40 kg/m^2^; Age: 25–60 years	ADF	35	CR	35	2
Pureza et al. (43)	Brazil	RBR-387v6v	BMI: 30–45 kg/m^2^	TRF+CR (12:12 diet)	31	CR	27	1
Razavi et al. (45)	Iran	NA	BMI: 25–40 kg/m^2^ with MetS; Age: 25–60 years	ADF	40	CR	40	4
Schübel et al. (46)	Germany	NCT02449148	BMI: 25–40 kg/m^2^; Age: 35–65 years	ICR (2:5 diet)	45	CR	49	12
						Non-intervention diet	52	
Stote et al. (47)	USA	NA	BMI: 18–25 kg/m^2^; Age: 40–50 years	TRF (20:4 diet)	15	Non-intervention diet	15	6
Teng et al. (49)	Malaysia	NA	BMI: 23–29.9 kg/m^2^; Age: 50–70 years	FCR (2:5 diet)	14	Non-intervention diet	14	3
Teng et al. (48)	Malaysia	NA	BMI: 23–29.9 kg/m^2^; Age: 50–70 years	FCR (2:5 diet)	28	Non-intervention diet	28	3
Tinsley et al. (50)	USA	NA	NA	TRF (20:4 diet + 4:3 diet)	14	Non-intervention diet	14	2
Tinsley et al. (51)	USA	NCT03404271	Age: 18–30 years	TRF (16:8 diet)	13	Non-intervention diet	14	2
Trabelsi et al. (9)	Tunisia	NA	NA	Ramadan	10	Non-intervention diet	9	1
Trabelsi et al. (52)	Tunisia	NA	NA	Ramadan	8	Non-intervention diet	8	1
Varady et al. (53)	USA	NA	BMI: 20–29.9 kg/m^2^; Age: 35–65 years	ADF	16	Non-intervention diet	16	3
Zouhal et al. (54)	Tunisia	NA	BMI: 30–40 kg/m^2^	Ramadan	14	Non-intervention diet	14	1

*RCT, randomized controlled trial; IER, intermittent energy restriction; ADF, alternate-day fasting; DER, daily energy restriction; TRF, time-restricted feeding; NAFLD, nonalcoholic fatty liver disease; IF, intermittent fasting; CR, calorie restriction; MED, Mediterranean diet; MetS, metabolic syndrome; ICR, intermittent calorie restriction; FCR, Muslim sunnah fasting; UK, United Kingdom; USA, the United States; BMI, body mass index; NA, no available; CER, continuous energy restriction; ICER, intermittent energy and carbohydrate restriction; DASH, Dietary Approaches to Stop Hypertension; 2:5 diet, 2-day fasting per week; 16:8 diet, 16-h fasting per day; 20:8 diet, 20-h fasting per day; 18:6 diet, 18-h fasting per day; 3:4 diet, 3-day fasting per week; 12:12 diet, 12-h fasting per day; 4:3 diet, 4-day fasting per week; N, number of patients*.

### Risk of Bias

Quality assessment of studies included is shown in supplementary Table 1. The Cochrane Collaboration's tool was used to assess the risk of bias in studies. Most studies were at low risk in sequence generation and allocation concealment. Only one study showed unclear risk in sequence generation due to incomplete information. As for blinding of the risk of bias, 10 trials were evaluated as high, 15 were unclear, and the rest 18 studies were low. Notably, 43 studies were at low risk for incomplete outcome data and selective outcome reporting. As for free of other bias, one was at high risk, 15 were at unclear risk, and the remaining 27 were at low risk.

### IF vs. Non-intervention Diet

**Figure 2:**
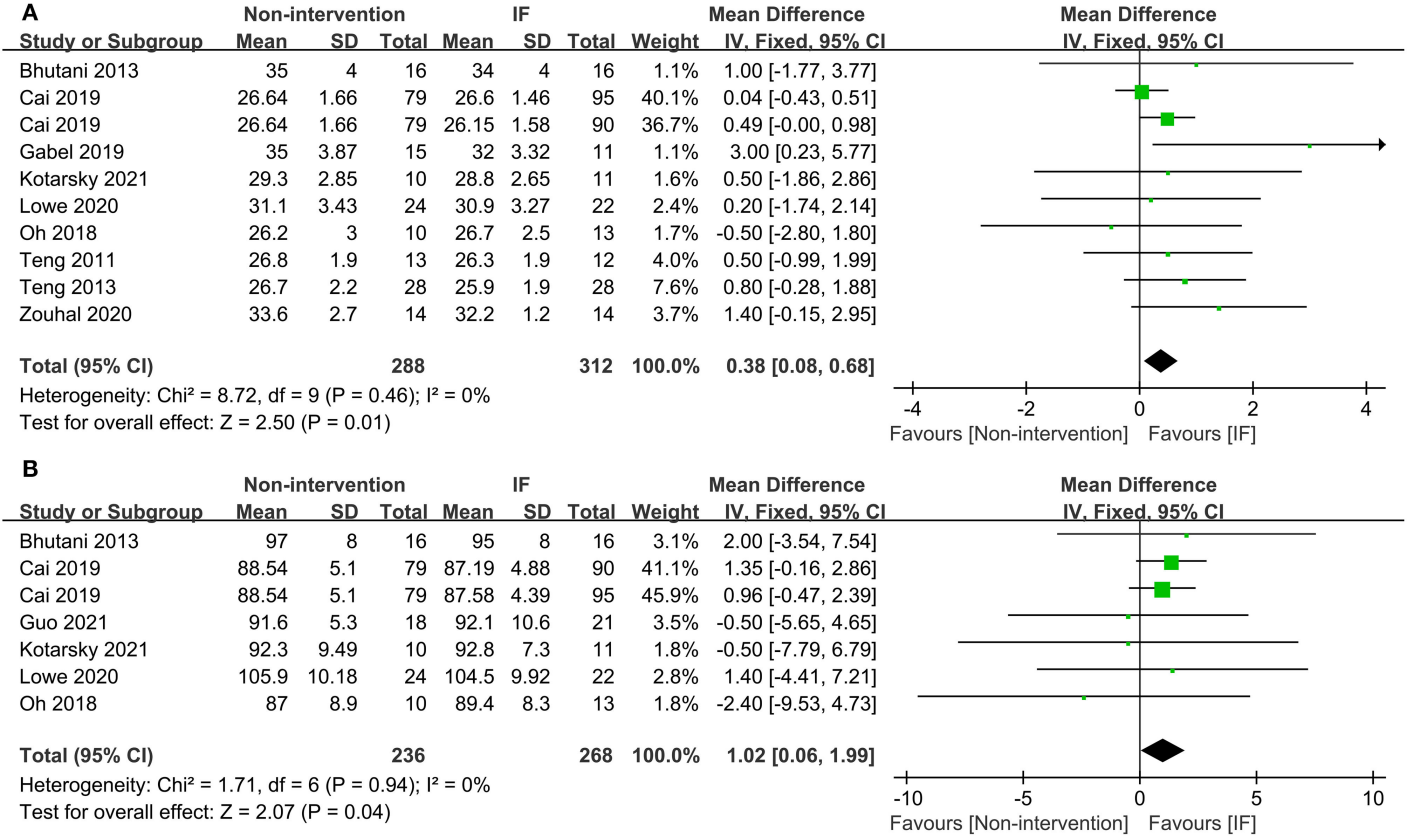
Forest plots for the body mass index (BMI) and waist circumference (WC) of intermittent fasting (IF) vs. non-intervention diet. **(A)** BMI; **(B)** WC.

Nineteen studies reported the weight of participants after IF and for those who maintained the non-intervention diet. The results illustrated that IF induced greater weight loss (WMD = 1.10, 95% CI: 0.09–2.12, *p* = 0.03) (supplementary Table 2). For BMI, the collective analysis of BMI as a result of following the IF diets resulted in greater reductions in BMI compared to the BMI following habitual diets (WMD = 0.38, 95% CI: 0.08–0.68, *p* = 0.01) (Figure 2A). In addition, participants who received IF had smaller WC after the experiment compared to those who had undergone the non-intervention diet (WMD = 1.02, 95% CI: 0.06-1.99, *p* = 0.04) (Figure 2B).

**Figure 3:**
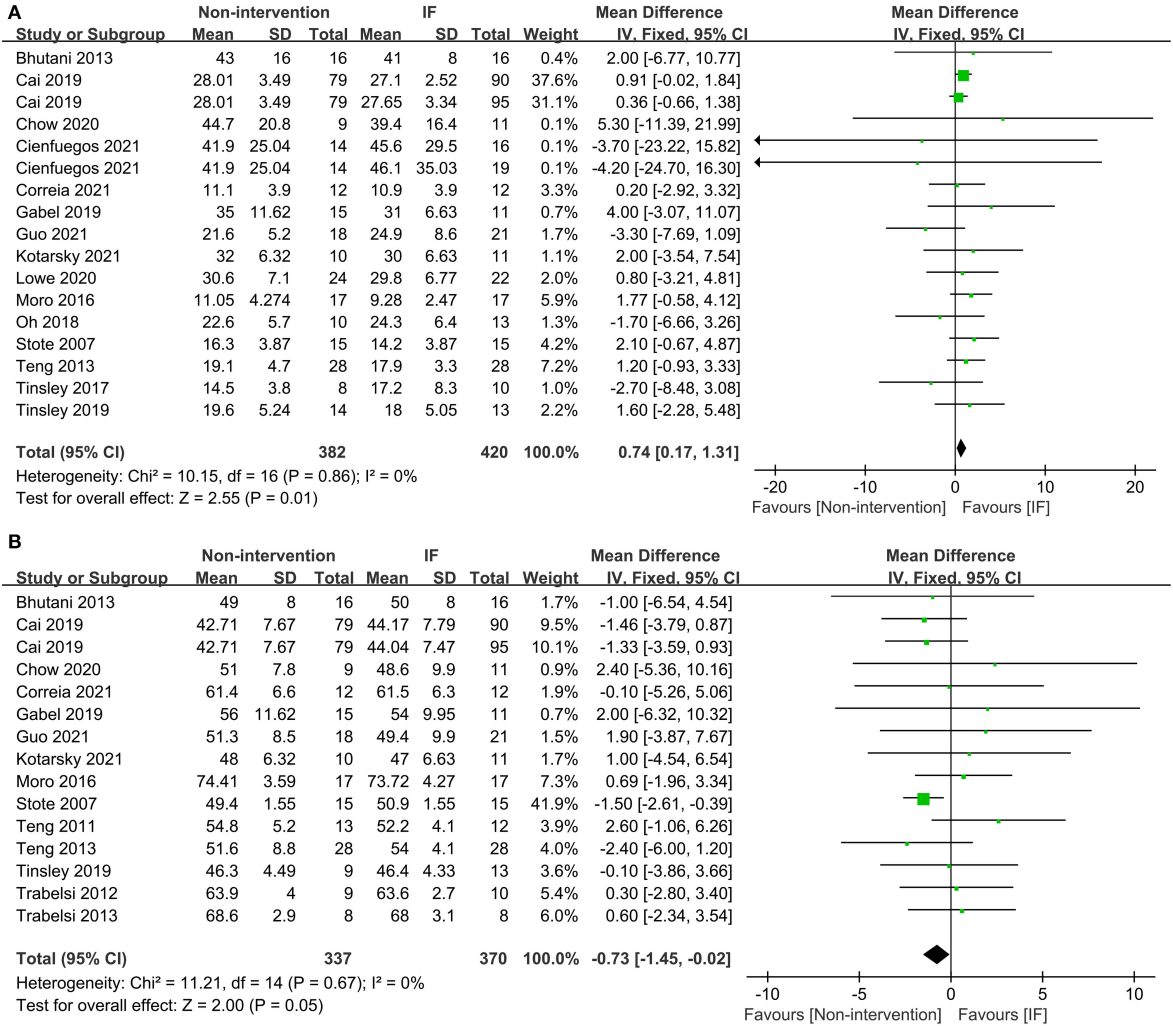
Forest plots for the fat mass (FM) and fat-free mass (FFM) of the IF vs. non-intervention diet. **(A)** FM; **(B)** FFM.

In terms of body composition, the participants in the IF group had less FM than those in the non-intervention diet group after interventions (WMD = 0.74, 95% CI: 0.17–1.31, *p* = 0.01) (Figure 3A). The fat-free mass (FFM) of participants who underwent IF was greater than that of those in the non-intervention diet group, demonstrating that IF had less effect on the FFM (WMD = −0.73, 95% CI: (−1.45)–(−0.02), *p* = 0.05) (Figure 3B). However, for FM percentage (FM%) after the intervention, there was no significant difference between IF and non-intervention diet groups (WMD = 0.38, 95% CI: (−0.14)−0.89, *p* = 0.16) (supplementary Table 2). Ten studies provided data on systolic blood pressure (SBP) and diastolic blood pressure (DBP) of the participants after the intervention. The results revealed that neither SBP nor DBP was affected *via* IF or non-intervention diet (WMD = 1.32, *p* = 0.33, and WMD = 0.96, *p* = 0.39, respectively) (supplementary Table 2).

**Figure 4:**
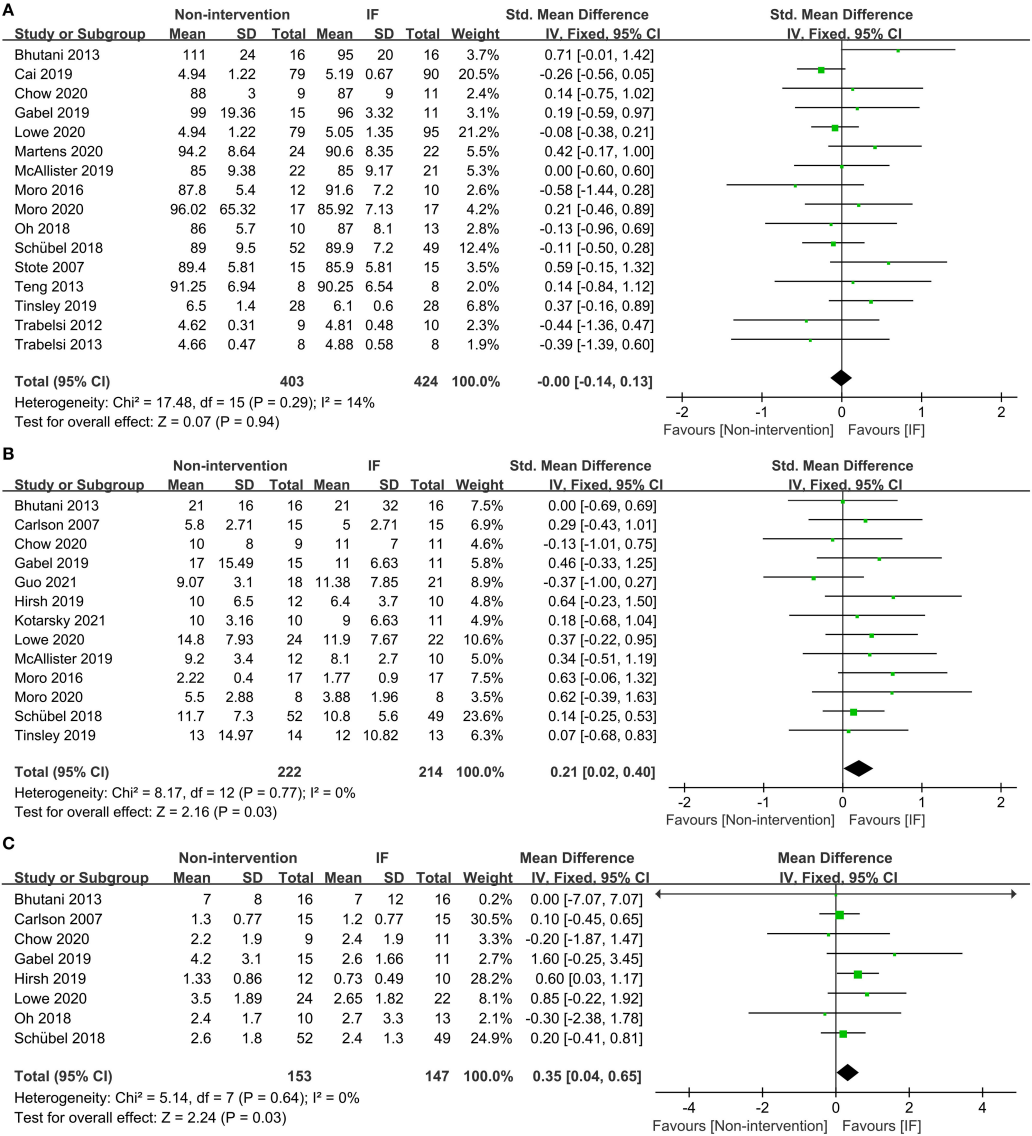
Forest plots for the glucose, insulin, and homeostasis model assessment insulin resistance (HOMA-IR) of IF vs. non-intervention diet. **(A)** Glucose; **(B)** insulin; **(C)** HOMA-IR.

Sixteen studies examined the effects of IF and the non-intervention diet on the fasting glucose concentrations of participants, where no difference was observed (SMD = −0.00, 95% CI: (−0.14)−0.13, *p* = 0.94) (Figure 4A). Thirteen studies investigated the effects of IF and non-intervention diet on insulin concentrations in participants. The correlated systematic analysis showed that the insulin levels after IF were significantly lower than those after the non-intervention diet (SMD = −0.21, 95% CI: 0.02–0.40, *p* = 0.03) (Figure 4B). In addition, the result of homeostasis model assessment insulin resistance (HOMA-IR) from 8 studies showed that IF could reduce insulin resistance more effectively compared to the non-intervention diet (WMD = 0.35, 95% CI: 0.04–0.65, *p* = 0.03; *I*^2^ = 0%) (Figure 4C).

**Figure 5:**
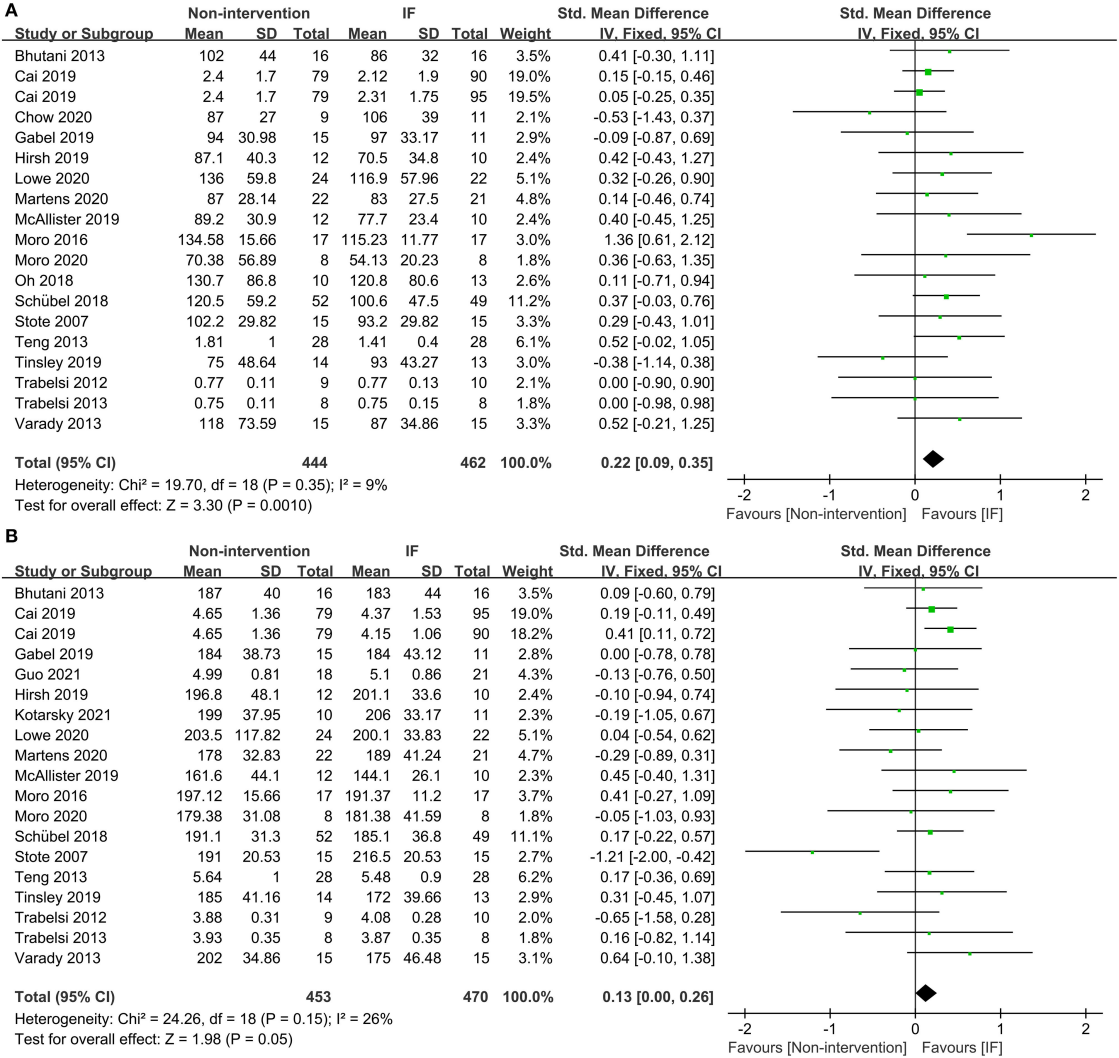
Forest plots for the triglyceride (TG) and total cholesterol (TC) of IF vs. non-intervention diet. **(A)** TG; **(B)** TC.

Eighteen studies provided the fasting TG concentrations of participants following IF and the non-intervention diet, and IF was proved to be significantly more effective in reducing TG in participants (SMD = 0.22, 95% CI: 0.09–0.35, *p* = 0.001) (Figure 5A). Eighteen studies reported the TC concentrations in IF group and non-intervention diet group, and the results indicated that participants had significantly lower TC in the former case (SMD = 0.13, 95% CI: 0.00–0.26, *p* = 0.05; *I*^2^ = 26%) (Figure 5B). In addition, 17 and 16 studies reported statistical results for low-density lipoprotein (LDL) and high-density lipoprotein (HDL), respectively. The statistical difference of those parameters between groups of IF and non-intervention diet was discovered in neither aspect (SMD = 0.10, *p* = 0.42, and SMD = −0.03, *p* = 0.63, respectively) (supplementary Table 2).

### IF vs. CR

The physical and biochemical parameters of IF vs. CR were analyzed in this study. The result of 10 studies showed that IF reduced participants' WC more than CR. The result was statistically significant with low heterogeneity (WMD = 2.29, 95% CI: 0.57–4.01, *p* = 0.009; *I*^2^ = 35%). Moreover, no difference was observed between IF and CR in terms of weight, glucose, and blood lipids between groups. Details of the results are shown in Table 2.

**Table 2:** Physical and biochemical parameters of IF vs. CR.

	**No. of studies**	**SMD/WMD**	**95%CI**	***p***	**I^**2**^ (%)**	**Effect-model**
Weight	6	2.08	−0.61–4.77	0.13	22	Fixed
BMI	11	0.35	−0.64–1.34	0.49	57	Random
WC	10	2.29	0.57–4.01	**0.009**	35	Fixed
FM(kg)	12	1.96	−0.34–4.27	0.09	72	Random
FM%	8	0.77	−0.62–2.16	0.28	5	Fixed
FFM(kg)	10	0.65	−0.51–1.81	0.27	0	Fixed
SBP	9	1.75	−0.41–3.90	0.11	45	Fixed
DBP	9	1.06	−0.53–2.65	0.19	0	Fixed
Glucose	12	−0.01	−0.16–0.14	0.87	22	Fixed
Insulin	10	0.07	−0.09–0.22	0.40	44	Fixed
HOMA-IR	7	0.00	−0.31–0.32	0.99	54	Random
TG	10	0.03	−0.14–0.19	0.76	20	Fixed
TC	10	0.08	−0.09–0.24	0.35	0	Fixed
LDL	9	0.06	−0.11–0.23	0.49	0	Fixed

*CR, calorie restriction; IF, intermittent fasting; BMI: body mass index; WC, waist circumference; FM, fat mass; FFM, fat-free mass; SBP, systolic blood pressure; DBP, diastolic blood pressure; HOMA-IR, homeostasis model assessment insulin resistance; TC, total cholesterol; TG, triglyceride; LDL, low-density lipoprotein; SMD, standard mean difference; WMD, weighted mean difference; CI, confidence interval. SMD: glucose, insulin, TG, TC, and LDL; WMD: weight, BMI, WC, FM (kg), FM%, FFM (kg), SBP, DBP, and HOMA-IR*.

*Bold indicates statistically significant differences (P < 0.05)*.

### Subgroup Analysis

The main pattern of IF in this study was ADF and weekly TRF, so they were compared with CR separately. The physical and biochemical parameters of participants in ADF and CR groups that were included in the investigation were weight, body composition, glucose, and blood lipids. According to the statistical results, there was no significant difference in all parameters between the two groups. In other words, the effects of ADF and CR on participants were similar (Table 3). In addition, 8 studies compared post-intervention data of weekly TRF vs. CR. The statistical result of WC extracted from 6 studies showed that weekly TRF reduced WC more effectively than CR (WMD = 3.36, 95% CI: 1.24–6.08, *p* = 0.003; *I*^2^ = 38%) (Table 3). Five studies reported the FM of the weekly TRF and CR subgroup and more reduced FM was discovered in the weekly TRF group (WMD = 2.58, 95% CI: 0.61–4.55, *p* = 0.01) (Table 3). Weekly TRF was also more effective in reducing DBP in participants (WMD = 3.47, 95% CI: (−0.06)−7.00, *p* = 0.05) (Table 3). More detailed physical and biochemical results are recorded in Table 3.

**Table 3:** Physical and biochemical parameters of ADF vs. CR and TRF (week) vs. CR.

	**No. of studies**	**SMD/WMD**	**95%CI**	***p***	**I^**2**^ (%)**	**Effect-model**
**ADF vs. CR**						
Weight	6	2.09	−2.91–7.10	0.41	63	Random
BMI	5	0.66	−0.24–1.57	0.15	45	Fixed
WC	3	1.37	−1.34–4.08	0.32	28	Fixed
FM(kg)	6	2.80	−0.74–6.34	0.12	78	Random
FM%	3	1.11	−1.00–3.23	0.30	38	Fixed
FFM(kg)	4	0.23	−1.64–2.11	0.81	0	Fixed
SBP	3	4.34	−5.83–14.50	0.40	79	Random
DBP	3	−0.03	−2.47–2.41	0.98	0	Fixed
Glucose	4	0.10	−0.32–0.53	0.63	56	Random
Insulin	4	0.25	−0.23–0.73	0.31	65	Random
TG	4	−0.01	−0.25–0.24	0.96	28	Fixed
TC	4	0.04	−0.21–0.28	0.78	0	Fixed
LDL	4	0.05	−0.19–0.30	0.68	0	Fixed
HDL	4	−0.08	−0.59–0.44	0.77	71	Random
**TRF(week) vs. CR**						
Weight	8	2.41	−0.33–5.14	0.08	42	Fixed
BMI	4	0.66	−0.91–2.22	0.41	51	Random
WC	6	3.66	1.24–6.08	**0.003**	38	Fixed
FM(kg)	5	2.58	0.61–4.55	**0.01**	45	Fixed
FM%	4	1.42	−0.68–3.53	0.18	0	Fixed
FFM(kg)	5	1.27	−0.29–2.82	0.11	2	Fixed
SBP	4	3.19	−1.26–7.63	0.16	0	Fixed
DBP	4	3.47	−0.06–7.00	**0.05**	0	Fixed
Glucose	6	−0.08	−0.29–0.14	0.49	14	Fixed
Insulin	4	0.05	−0.34–0.43	0.81	59	Random
TG	5	0.11	−0.14–0.36	0.39	33	Fixed
TC	4	0.13	−0.13–0.40	0.33	0	Fixed
LDL	4	0.07	−0.20–0.33	0.63	0	Fixed
HDL	4	−0.07	−0.34–0.19	0.60	0	Fixed

*CR, calorie restriction; ADF, alternate-day fasting; TRF, time-restricted feeding; BMI: body mass index; WC, waist circumference; FM, fat mass; FFM, fat-free mass; SBP, systolic blood pressure; DBP, diastolic blood pressure; TC, total cholesterol; TG, triglyceride; LDL, low-density lipoprotein; HDL, high-density lipoprotein; SMD, standard mean difference; WMD, weighted mean difference; CI, confidence interval. SMD: glucose, insulin, TG, TC, LDL, and HDL; WMD: weight, BMI, WC, FM (kg), FM%, FFM (kg), SBP, and DBP*.

*Bold indicates statistically significant differences (P < 0.05)*.

Subgroup analysis found that, compared with non-intervention diet and CR, IF had different effects depending on sex. When it comes to women, IF was significantly more effective with highly heterogeneous in reducing FM (WMD = 3.72, 95% CI: 0.56–6.87, *p* = 0.02; *I*^2^ = 67%) (Table 4). However, no advantage was discovered in any other aspects, such as weight, WC, FM%, FFM, fasting glucose, and insulin level. As for men, the statistical results showed that IF could significantly reduce men's weight and BMI compared with both non-intervention diet and CR, which were supported by 8 and 4 studies, respectively (WMD = 2.16, 95% CI: 0.48–3.85, *p* = 0.01; *I*^2^ = 29% and WMD = 0.97, 95% CI: 0.23–1.71, *p* = 0.01; *I*^2^ = 0%, respectively) (Table 4). Moreover, IF also reduced TG levels more effectively in men (WMD = 0.54, 95% CI: 0.22–0.86, *p* < 0.001; *I*^2^ = 31%). More detailed physical and biochemical results are listed in Table 4.

**Table 4:** Physical and biochemical parameters of IF vs. non-intervention diet/CR in women and men.

	**No. of studies**	**SMD/>WMD**	**95%CI**	***p***	**I^**2**^ (%)**	**Effect–model**
**Female**						
Weight	6	2.08	−0.61–4.77	0.13	22	Fixed
WC	5	1.80	−1.53–5.13	0.29	50	Random
FM(kg)	5	3.72	0.56–6.87	**0.02**	67	Random
FM%	3	0.68	−1.45–2.80	0.53	0	Fixed
FFM(kg)	4	1.10	−0.37–2.57	0.14	0	Fixed
Glucose	3	−0.00	−0.17–0.17	1.00	0	Fixed
Insulin	3	0.92	−0.21–2.06	0.11	0	Fixed
**Male**						
Weight	8	2.16	0.48–3.85	**0.01**	29	Fixed
BMI	4	0.97	0.23–1.71	**0.01**	0	Fixed
FM(kg)	4	0.98	−0.38–2.35	0.16	0	Fixed
FM%	7	0.40	−0.12–0.92	0.14	0	Fixed
FFM(kg)	6	0.37	−0.98–1.72	0.59	0	Fixed
Glucose	6	0.09	−0.21–0.40	0.55	0	Fixed
TG	6	0.54	0.22–0.86	**<0.001**	31	Fixed
TC	6	0.15	−0.16–0.45	0.36	0	Fixed
LDL	5	0.17	−0.16–0.49	0.32	19	Fixed
HDL	5	−0.09	−0.41–0.24	0.60	0	Fixed

*CR, calorie restriction; IF, intermittent fasting; BMI: body mass index; WC, waist circumference; FM, fat mass; FFM, fat-free mass; TC, total cholesterol; TG, triglyceride; LDL, low-density lipoprotein; HDL, high-density lipoprotein; SMD, standard mean difference; WMD, weighted mean difference; CI, confidence interval. SMD: glucose (men), TG, TC, and LDL; WMD: weight, BMI, WC, FM (kg), FM%, FFM (kg), glucose (women), and insulin*.

*Bold indicates statistically significant differences (P < 0.05)*.

According to the International Obesity Task Force (IOTF), a BMI of 25–30 units in adults is defined as being overweight, while a BMI greater than 30 in an individual is considered to be obese (1). Herein, subgroup analysis was performed specifically on patients with overweight or obesity (BMI ≥ 25 kg/m^2^) among the included participants. The patients' physical and biochemical parameters of the IF vs. non-intervention diet are recorded in Table 5. The results showed that IF could significantly reduce insulin resistance compared with the non-intervention diet (HOMA-IR: WMD = 0.43, 95% CI: 0.04–0.83, *p* = 0.03). However, there was no significant difference in weight, body composition, and blood lipids. Moreover, the effectiveness of IF and CR was similar in patients with overweight or obesity. The correlated physical and biochemical parameters are recorded in Table 6.

**Table 5:** Physical and biochemical parameters of IF vs. non-intervention diet in patients with BMI ≥ 25 kg/m^2^.

	**No. of studies**	**WMD**	**95%CI**	***p***	**I^**2**^ (%)**	**Effect–model**
Weight	6	3.42	−0.77–7.61	0.11	0	Fixed
BMI	3	1.39	−0.12–2.90	0.07	0	Fixed
FM(kg)	5	2.52	−1.22–6.26	0.19	0	Fixed
FFM(kg)	4	0.72	−2.51–3.94	0.66	0	Fixed
SBP	5	1.63	−3.12–6.37	0.50	0	Fixed
DBP	5	0.65	−2.52–3.81	0.69	7	Fixed
Glucose	4	0.33	−2.36–3.02	0.81	39	Fixed
Insulin	6	1.46	−0.36–3.29	0.12	0	Fixed
HOMA–IR	5	0.43	0.04–0.83	**0.03**	0	Fixed
TG	5	7.70	−3.85–19.26	0.19	33	Fixed
TC	5	2.81	−7.36–12.99	0.59	0	Fixed
LDL	5	4.73	−3.32–12.79	0.25	0	Fixed
HDL	6	0.40	−3.06–3.86	0.82	49	Fixed

*IF, intermittent fasting; BMI: body mass index; FM, fat mass; FFM, fat-free mass; SBP, systolic blood pressure; DBP, diastolic blood pressure; HOMA-IR, homeostasis model assessment insulin resistance; TC, total cholesterol; TG, triglyceride; LDL, low-density lipoprotein; HDL, high-density lipoprotein; WMD, weighted mean difference; CI, confidence interval*.

*Bold indicates statistically significant differences (P < 0.05)*.

**Table 6:** Physical and biochemical parameters of IF vs. CR in patients with BMI ≥25 kg/m^2^.

	**No. of studies**	**SMD/WMD**	**95%CI**	***p***	**I^**2**^ (%)**	**Effect-model**
Weight	13	0.25	−2.88–3.37	0.88	52	Random
BMI	11	0.35	−0.64–1.34	0.49	57	Random
WC	7	1.58	−0.60–3.76	0.16	36	Fixed
FM(kg)	9	1.61	−1.32–4.54	0.28	74	Random
FM%	6	0.54	−1.13–2.21	0.53	26	Fixed
FFM(kg)	8	−0.13	−1.70–1.44	0.87	0	Fixed
SBP	9	1.75	−0.41–3.90	0.11	45	Fixed
DBP	9	1.06	−0.53–2.65	0.19	0	Fixed
Glucose	10	0.05	−0.12–0.23	0.55	16	Fixed
Insulin	9	0.02	−0.15–0.19	0.82	42	Fixed
HOMA-IR	6	−0.18	−0.39–0.03	0.10	46	Fixed
TG	10	0.03	−0.14–0.19	0.76	20	Fixed
TC	9	0.08	−0.09–0.25	0.35	0	Fixed
LDL	9	0.06	−0.11–0.23	0.49	0	Fixed
HDL	9	0.02	−0.15–0.19	0.79	42	Fixed

*CR, calorie restriction; IF, intermittent fasting; BMI: body mass index; WC, waist circumference; FM, fat mass; FFM, fat-free mass; SBP, systolic blood pressure; DBP, diastolic blood pressure; HOMA-IR, homeostasis model assessment insulin resistance; TC, total cholesterol; TG, triglyceride; LDL, low-density lipoprotein; HDL, high-density lipoprotein; SMD, standard mean difference; WMD, weighted mean difference; CI, confidence interval. SMD: glucose, insulin, TG, TC, LDL, and HDL; WMD: weight, BMI, WC, FM (kg), FM%, FFM (kg), SBP, DBP, and HOMA-IR*.

### Sensitivity Analysis and Publication Bias Analysis

After sensitive analysis, we found that the results were stable. Meanwhile, funnel plots were used to examine publication bias. The funnel plots of all statistical results were displayed to be roughly symmetry, indicating that there was no publication bias.

## Discussion

At present, more and more people in the world are suffering from metabolic diseases and obesity (1). Although IF was considered to be related to undesired outcomes, such as gout, arrhythmia, and peptic ulcer (55), it is still widely believed to be able to reduce weight, relive rheumatoid arthritis, and slow down aging (56, 57), even if they still disagreed on cancer (58, 59). In this study, IF was compared with a non-intervention diet and CR, respectively. In addition, subgroup analysis was conducted based on the pattern of IF, sex, and specific population, which narrowed the population within the overweight or obese population.

In our study, IF was found to have significant benefits in improving outcome indices for weight, FM, insulin, and blood lipids compared to a non-intervention diet. These results were similar to previous studies (6, 56, 60), indicating that IF can result in desirable weight loss and better health. However, there was no difference in fasting blood glucose in participants between the IF group and the non-intervention diet group in this study. It might be due to the fact that the included population in our analysis was mostly non-diabetic individuals, so the basal glucose concentrations were relatively low, resulting in slight fluctuations. As for the results of insulin concentrations and HOMA-IR, IF was proved to be potentially beneficial in relieving insulin resistance. In addition, during the analysis of our study, it was found that in some studies, participants' IF was accompanied by physical exercise. The combination may be more conducive to improving participants' health than IF alone (40). But subjects in both intervention and control groups in the same RCT performed exercise or neither, so the effect of physical activity on this analysis was controllable. In this study, IF was about as effective as CR, only slightly better than CR in reducing WC. Although some researchers believed that, compared with CR, IF had better compliance in participants and was more beneficial in reducing FM (11, 57), other studies showed that long-term compliance of IF was limited due to the high dropout rate (in contrast to CR, IF had a 38% dropout rate) (61, 62).

As for the comparison between ADF and CR, the subgroup analysis based on the dietary pattern of IF found that ADF had no greater beneficial effects than CR in our study, but other studies showed a difference in superior compliance, FM, and FFM in the ADF group (11, 63). Furthermore, ADF also did not have the burden of chronic poor feeding and other adverse outcomes compared to CR (58). However, some investigators believed that ADF might not be a viable public health intervention because of considerably and continuously reported hunger by Patterson et al. (8, 64). It was believed that hunger affected participants' enthusiasm and adherence to fasting (64). In addition, compared to ADF, weekly TRF exerted significant advantages on the regulation of WC, FM, and DBP. Another meta-analysis also reported that besides weight, DBP, and insulin regulation, TRF was also more effective in reducing FFM (12).

In addition, IF might play a small role in women because FM was the only parameter that was found to be significantly reduced after IF in women. Comparatively, IF was found to have significantly reduced weight and TG in men. The reason for the difference based on sex was currently not clear. It may be due to the differences in energy intake between men and women, but this study was not able to qualitatively compare the energy intake between men and women because of different RCT settings with different energy limits. Besides, we also assumed that it might be related to the sex-depending fat distribution and sex hormones (65). Estrogen was considered to be able to suppress appetite and reduce the accumulation of belly fat, while androgen promoted food intake. However, research on the relationship between IF and human sex hormones is lacking. Moreover, our subgroup analysis against patients with overweight or obesity showed that IF could not provide more benefit to this particular population. This was at odds with previous studies by researchers who believed that IF is functional in regulating blood pressure, TC, and TG, especially more than CR does, in patients with overweight or obesity (30, 66). One theory we speculated was the insufficient quantity of included studies, another might be that the intervention time was relatively short in our study, which was a median follow-up of only 3 months, thus no appearance of IF efficacy was observed.

In many ways, the effects of IF and CR overlapped significantly (55). The essence of both IF and CR was to reduce energy intake. The difference was that CR maintained a normal eating frequency, while IF was no or small amounts of energy intake during fasting (58). Although most of the included studies in this study did not perform energy intake interventions during non-fasting periods, there were still some studies that restricted energy intake during feeding periods, and certain eating patterns of IF also shaped a certain degree of energy restriction (18, 48). Thus, while IF was discovered to be superior to the non-intervention diet in this study, it was unclear whether time restriction or energy restriction played a greater role. In contrast, in our study, IF and CR had few differences in their effects on participants, while the previous studies showed that CR could promote weight loss, relieve insulin resistance, improve insulin sensitivity, lower TG and TC, and elevate HDL (67). Although IF did not restrict calories in some of the included studies (36), it is suspected in this study that CR may be dominant as time restriction did not seem to play a role.

Some researchers suggested that weight loss after CR was due to the adaptation to metabolically induced reductions in FM and FFM (67, 68). The weight loss resulting from IF was also due to the reduction of FM and FFM. However, the difference was that IF, in addition to metabolic adaptations, consumed the stored hepatic glycogen 10–12 h after fasting and then generated massive ketone bodies through the oxidation of fatty acids in adipose tissue, which would be used as the energy source of the whole body (69, 70). Therefore, an increase in blood ketones was found after IF, but not after CR alone (55). Unfortunately, this investigation was not included in this study. The decrease in blood TG might also be due to the oxidative breakdown of fat and the restriction of fat intake (67). Although previous studies attributed weight loss after IF and CR to the reduction of FM and FFM (67, 70), it was an important discovery in this study that IF preserved FFM better. This finding contributes to further recognition of IF, which is that IF does not damage lean tissue, dissipating the concern of IF being the potential cause of osteoporosis and sarcopenia (12).

One of the neuroendocrine mechanisms by which CR mediated was that CR could cause decreased levels of anabolic hormones including insulin, leptin, estrogen, and testosterone (71). CR in combination with weight loss can further improve insulin sensitivity, which might lead to decreased insulin secretion (72). In addition to the above possible mechanisms, IF might also act on the central nervous system *via* ketone bodies generated by fat metabolism. This process enhances the effect of leptin and insulin on the central nervous system, which normally regulates food intake and insulin sensitivity as well as resistance (62). Herein, insulin levels decreased, and insulin resistance was relieved after IF with no significance. Both CR and IF lowered TC (67). This is probably due to the mechanistic target of the rapamycin (mTOR) pathway (55, 71). IF might lower TC by inhibiting TC production *via* suppressing this pathway. Since this pathway could also be activated by CR, it is reasonable to discover no difference in TC concentrations between the two intervention groups.

Researchers have proposed three possible mechanisms for IF, such as circadian rhythms, gastrointestinal microbiota, and modifiable lifestyle behaviors (8). Some researchers argued that the human body clock was affected by, but not limited to, metabolic hormones, nutrients, intracellular metabolism, and intestinal flora (73). The human circadian rhythms regulate eating, sleep, hormonal and physiological processes, coordinated metabolism, and energetics. Certain patterns of IF might affect the body clock by revising the time humans eat, thus realigning metabolism and energy allocation to ensure human health (8). Sutton's study of daily TRF, and 6-h daily eating during 5 weeks, showed that TRF improved cardio-metabolic health (74). Unfortunately, due to the limited number of studies, we were unable to conduct a comparative analysis between daily TRF and CR or a non-intervention diet. In contrast, the feeding window of the non-fasting period for weekly TRF and ADF was not addressed in the viewed RCTs (45, 75), so it was unknown whether circadian rhythms play a role in these two patterns of IF. An irregular and inappropriate diet might lead to metabolic disorders and other adverse consequences (73). Although current IF was actually accompanied by energy restriction, it was undeniable that IF limited daytime hours of feeding, which might contribute to improved parameters (8). Both the two other hypotheses, such as gastrointestinal microbiota and modifiable lifestyle behaviors, lacked human clinical trials and could not be confirmed in relation to IF in this study.

This study is a systematic meta-analysis based on RCTs of IF, and it is multi-angle and relatively comprehensive compared with previous meta-analyses (76–78). This study compared IF with CR and non-intervention diets separately, and subgroup analysis was performed according to the patterns of IF and population characteristics. However, there are still considerable limitations in this study. First, this study mixes various patterns of IF, which might reduce the reliability and interpretability. Second, part of the outcomes, such as hip circumference and waist-to-hip ratio, could not be analyzed in this study due to insufficient data, which might produce publication bias. Moreover, although subgroup analysis was conducted, the role of various factors such as the long-term effect of IF was not thoroughly analyzed in detail due to the limited number of studies. Finally, only studies that were published in English were included, which also may bring publication bias.

## Conclusion

In conclusion, our analysis revealed that IF was more beneficial in improving body weight, WC, and FM without affecting lean mass compared to a non-intervention diet. IF could also improve the condition of insulin resistance and blood lipid compared with non-intervention diets, but act similar to CR. Different patterns of IF had different effects on metabolism. Moreover, the effects of IF were not uniform across women and men or in the overweight or obese population. More and larger multicentered studies are needed to evaluate IF, while this study may lay a foundation for follow-up research to examine more extensively the reliable effects of IF.

## Data Availability Statement

The original contributions presented in the study are included in the article/Supplementary Material, further inquiries can be directed to the corresponding author/s.

## Author Contributions

HL and LG designed the research process. LG and RF searched the database for corresponding articles and drafted the meta-analysis. JH extracted useful information from the articles above. HN used statistical software for analysis. KY polished this article. All the authors had read and approved the manuscript and ensured that this was the case.

## Conflict of Interest

The authors declare that the research was conducted in the absence of any commercial or financial relationships that could be construed as a potential conflict of interest.

## Publisher's Note

All claims expressed in this article are solely those of the authors and do not necessarily represent those of their affiliated organizations, or those of the publisher, the editors and the reviewers. Any product that may be evaluated in this article, or claim that may be made by its manufacturer, is not guaranteed or endorsed by the publisher.

## Abbreviations

IF: intermittent fasting
ADF: alternate-day fasting
IER: intermittent energy restriction
TRF: time-restricted feeding
CR: caloric restriction
FM: fat mass
RCT: randomized controlled trial
BMI: body mass index
WC: waist circumference
TC: total cholesterol
TG: triglyceride
WMD: weighted mean difference
SMD: the standard mean difference
CI: confidence intervals
FFM: fat-free mass
SBP: systolic blood pressure
DBP: diastolic blood pressure
HOMA-IR: homeostasis model assessment insulin resistance
LDL: low-density lipoprotein
HDL: high-density lipoprotein.
