# Supplementary material for: Effects of Intermittent Fasting in Human Compared to a Non-intervention Diet and Caloric Restriction: A Meta-Analysis of Randomized Controlled Trials
Source: Front Nutr. 2022 May 2;9:871682. doi: 10.3389/fnut.2022.871682 (PMC9108547; doi:10.3389/fnut.2022.871682)
Supplement: Supplementary file 1 [file Table_1.DOCX]

**Supplementary Table 1.** Quality assessment of studies included.

| Author, year,  Study (RCT) | Sequence  Generation | Allocation  Concealment | Blinding | Incomplete  outcome data | Selective  outcome reporting | Free of  other bias |
| --- | --- | --- | --- | --- | --- | --- |
| Antoni, 2018 | Low risk | Low risk | Low risk | Low risk | Low risk | Low risk |
| Beaulieu, 2019 | Low risk | Low risk | Low risk | Low risk | Low risk | Low risk |
| Bhutani, 2013 | Low risk | Low risk | Low risk | Low risk | Low risk | High risk |
| Bowen, 2018 | Low risk | Low risk | High risk | Low risk | Low risk | Low risk |
| Cai, 2019 | Low risk | Low risk | Unclear risk | Low risk | Low risk | Low risk |
| Carlson, 2007 | Low risk | Low risk | Low risk | Low risk | Low risk | Unclear risk |
| Catenacci, 2016 | Unclear risk | Low risk | Low risk | Low risk | Low risk | Unclear risk |
| Chow, 2020 | Low risk | Low risk | Unclear risk | Low risk | Low risk | Low risk |
| Cienfuegos, 2021 | Low risk | Low risk | Low risk | Low risk | Low risk | Low risk |
| Conley, 2017 | Low risk | Low risk | Low risk | Low risk | Low risk | Low risk |
| Corley, 2018 | Low risk | Low risk | High risk | Low risk | Low risk | Low risk |
| Correia, 2021 | Low risk | Low risk | Low risk | Low risk | Low risk | Unclear risk |
| Coutinho, 2017 | Low risk | Low risk | Unclear risk | Low risk | Low risk | Low risk |
| de Oliveira Maranhão Pureza, 2021 | Low risk | Low risk | High risk | Low risk | Low risk | Low risk |
| Gabel, 2019 | Low risk | Low risk | Low risk | Low risk | Low risk | Low risk |
| Guo, 2020 | Low risk | Low risk | Low risk | Low risk | Low risk | Low risk |
| Harvie, 2011 | Low risk | Low risk | Unclear risk | Low risk | Low risk | Unclear risk |
| Harvie, 2013 | Low risk | Low risk | Low risk | Low risk | Low risk | Low risk |
| Headland, 2018 | Low risk | Low risk | Low risk | Low risk | Low risk | Low risk |
| Hirsh, 2019 | Low risk | Low risk | Unclear risk | Low risk | Low risk | Low risk |
| Kotarsky, 2021 | Low risk | Low risk | Low risk | Low risk | Low risk | Low risk |
| Kunduraci, 2020 | Low risk | Low risk | Low risk | Low risk | Low risk | Low risk |
| Lowe, 2020 | Low risk | Low risk | Unclear risk | Low risk | Low risk | Low risk |
| Martens, 2020 | Low risk | Low risk | Unclear risk | Low risk | Low risk | Low risk |
| Martínez-Rodríguez, 2021 | Low risk | Low risk | Unclear risk | Low risk | Low risk | Low risk |
| McAllister, 2019 | Low risk | Low risk | Unclear risk | Low risk | Low risk | Unclear risk |
| Moro, 2016 | Low risk | Low risk | High risk | Low risk | Low risk | Unclear risk |
| Moro, 2020 | Low risk | Low risk | High risk | Low risk | Low risk | Low risk |
| Oh, 2018 | Low risk | Low risk | Unclear risk | Low risk | Low risk | Low risk |
| Panizza, 2019 | Low risk | Low risk | Low risk | Low risk | Low risk | Low risk |
| Parvaresh, 2019 | Low risk | Low risk | Low risk | Low risk | Low risk | Low risk |
| Pureza, 2020 | Low risk | Low risk | High risk | Low risk | Low risk | Low risk |
| Razavi, 2020 | Low risk | Low risk | Low risk | Low risk | Low risk | Unclear risk |
| Schübel, 2018 | Low risk | Low risk | High risk | Low risk | Low risk | Low risk |
| Stote, 2007 | Low risk | Low risk | Unclear risk | Low risk | Low risk | Unclear risk |
| Teng, 2011 | Low risk | Low risk | Unclear risk | Low risk | Low risk | Unclear risk |
| Teng, 2013 | Low risk | Low risk | Unclear risk | Low risk | Low risk | Unclear risk |
| Tinsley, 2016 | Low risk | Low risk | Unclear risk | Low risk | Low risk | Unclear risk |
| Tinsley, 2019 | Low risk | Low risk | Low risk | Low risk | Low risk | Low risk |
| Trabelsi, 2012 | Low risk | Low risk | High risk | Low risk | Low risk | Unclear risk |
| Trabelsi, 2013 | Low risk | Low risk | High risk | Low risk | Low risk | Unclear risk |
| Varady, 2013 | Low risk | Low risk | Unclear risk | Low risk | Low risk | Unclear risk |
| Zouhal, 2020 | Low risk | Low risk | High risk | Low risk | Low risk | Unclear risk |

Risk of bias was assessed as “low risk”, “high risk” or “unclear risk”.
