# Supplementary material for: Effects of Intermittent Fasting in Human Compared to a Non-intervention Diet and Caloric Restriction: A Meta-Analysis of Randomized Controlled Trials
Source: Front Nutr. 2022 May 2;9:871682. doi: 10.3389/fnut.2022.871682 (PMC9108547; doi:10.3389/fnut.2022.871682)
Supplement: Supplementary file 2 [file Table_2.DOCX]

**Supplementary Table 2.** Physical and biochemical parameters of IF versus non-intervention diet.

|  | No. of studies | SMD/WMD | 95%CI | *p* | I^2^ (%) | Effect-model |
| --- | --- | --- | --- | --- | --- | --- |
| Weight | 19 | 1.10 | 0.09-2.12 | **0.03** | 0 | Fixed |
| FM% | 8 | 0.38 | -0.14-0.89 | 0.16 | 0 | Fixed |
| SBP | 10 | 1.32 | -1.32-3.96 | 0.33 | 0 | Fixed |
| DBP | 10 | 0.96 | -1.21-3.13 | 0.39 | 24 | Fixed |
| LDL | 17 | 0.10 | -0.14-0.33 | 0.42 | 62 | Random |
| HDL | 16 | -0.03 | -0.17-0.10 | 0.63 | 0 | Fixed |

IF, intermittent fasting; FM, fat mass; SBP, systolic blood pressure; DBP, diastolic blood pressure; SMD, standard mean difference; SMD, standard mean difference; WMD, weighted mean difference; CI, confidence interval.

SMD: LDL, HDL; WMD: Weight, FM%, SBP, DBP.
